# A protocol for monitoring fidelity of a preconception-life course intervention in a middle-income setting: the Healthy Life Trajectories Initiative (HeLTI), South Africa

**DOI:** 10.1186/s13063-022-06696-w

**Published:** 2022-09-06

**Authors:** Larske M. Soepnel, Catherine E. Draper, Khuthala Mabetha, Cindy-Lee Dennis, Alessandra Prioreschi, Stephen Lye, Shane A. Norris

**Affiliations:** 1 SAMRC/Wits Developmental Pathways for Health Research Unit, Department of Paediatrics, Faculty of Health Sciences, School of Clinical Medicine, University of the Witwatersrand, Private Bag 3, Wits, Johannesburg, 2050 South Africa; 2 Julius Global Health, Julius Center for Health Sciences and Primary Care, University Medical Center Utrecht, Utrecht University, Utrecht, The Netherlands; 3 Lawrence S Bloomberg Faculty of Nursing, University of Toronto, Toronto, ON Canada; 4 Lunenfeld-Tanenbaum Research Institute, Sinai Health System, Department of Obstetrics and Gynecology, Department of Physiology and Medicine, University of Toronto, Toronto, ON Canada; 5 School of Human Development and Health, University of Southampton, Southampton, UK

**Keywords:** Fidelity, Protocol, Behavioural intervention, Low- and middle-income setting

## Abstract

**Introduction:**

Despite the importance of intervention fidelity in interpreting the outcomes of complex public health interventions, there is a lack of both reporting fidelity trial protocols and uniformity. In evaluating complex, adaptable/pragmatic interventions in resource-strapped settings with systemic issues, unique challenges to intervention adherence and monitoring are introduced, increasing the importance of a fidelity protocol. We aim to describe the intervention fidelity and monitoring protocol for the Healthy Life Trajectories Initiative (HeLTI) South Africa, a complex four-phase intervention set in urban Soweto, starting preconceptionally and continuing through to pregnancy, infancy, and early childhood to improve the health of young women and reduce the intergenerational risk of obesity.

**Methods:**

The HeLTI SA fidelity protocol was based on the NIH Behaviour Change Consortium (NIH BCC) Treatment Fidelity Framework, outlining the following components of intervention fidelity: study design, provider training, intervention delivery, intervention receipt, and intervention enactment. Context-specific fidelity challenges were identified. The intervention fidelity components and associated monitoring strategies were developed to align with HeLTI SA. Strategies for fidelity monitoring include, amongst others, qualitative process evaluation methods, reviewing observed and recorded intervention sessions, monitoring of activity logs, standardized training, and intervention session checklists. Possible challenges to fidelity and fidelity monitoring include high provider turnover, lack of qualification amongst providers, difficulty tracing participants for follow-up sessions, participant health literacy levels, and the need to prioritize participants’ non-health-related challenges. Solutions proposed include adapting intervention delivery methods, recruitment methods, and provider training methods.

**Discussion:**

The NIH BCC Treatment Fidelity Framework provided a solid foundation for reporting intervention fidelity across settings to improve intervention validity, ability to assess intervention effectiveness, and transparency. However, context-specific challenges to fidelity (monitoring) were identified, and transparency around such challenges and possible solutions in low- and middle-income settings could help foster solutions to improve adherence, reporting, and monitoring of intervention fidelity in this setting.

**Trial registration:**

Pan African Clinical Trials Registry PACTR201903750173871. Registered on 27 March 2019

**Supplementary Information:**

The online version contains supplementary material available at 10.1186/s13063-022-06696-w.

## Administrative information

Administrative information for HeLTI SA (*Bukhali*) Trial

**Table:** 

**Primary sponsor**	South African Medical Research Council Developmental Pathways for Health Research Unit University of the Witwatersrand**;** Chris Hani Baragwanath Hospital, 26 Chris Hani Road, Soweto, Johannesburg, South Africa, Pincode:1864
**Principal investigator**	Prof. Shane Norris; Prof. Stephen Lye
**Trial registration**	Pan African Clinical Trials Registry (https://pactr.samrc.ac.za/TrialDisplay.aspx?TrialID=6015; identifier: PACTR201903750173871, Registered 27 March 2019)

## Background

Intervention fidelity refers to the degree to which an intervention is delivered as initially planned. It functions as a moderating factor between the planned intervention and trial outcomes [1, 2]. It is therefore important to assess fidelity to be able to draw firm and confident conclusions about the degree of effectiveness of theory-based interventions, reducing type I and type II errors [3]. For complex behaviour and public health interventions, there is an increased likelihood that fidelity could be compromised. Therefore, a plan to monitor fidelity and anticipate challenges should be integrated into the intervention design and implementation.

With increasing interest in process evaluation for complex intervention studies, reporting of fidelity has become more common [1]. However, reporting still varies widely between studies, including those conducted in low- and middle-income settings. A recent systematic review of fidelity assessment protocols and trial reports of public health interventions in low- and middle-income countries (LMICs) found a lack of systematic fidelity assessment, with only 40% of protocols reporting any fidelity assessment plans, and most trial reports only assessing one or two dimensions of fidelity [4]. In another systematic review of interventions to improve community health worker performance in LMICs, no information on fidelity was provided by the 14 included studies [5]. Systematic reviews of fidelity assessment of behaviour change interventions in high-income settings have similarly found considerable heterogeneity in fidelity assessment approaches across studies, with few reporting on all relevant fidelity domains [6–8]. This lack of uniformity in fidelity reporting widens the gap between research and effective implementation into practice [9].

The Healthy Life Trajectories Initiative (HeLTI) South Africa (SA) is one site of the multinational HeLTI consortium, with complementary trials ongoing in Canada, India, and China, in collaboration with the World Health Organization. HeLTI aims to evaluate the cumulative effect of a complex package of interventions starting in the preconception period, through pregnancy and early childhood on childhood obesity as the primary outcome [10, 11]. Obesity and non-communicable diseases are rapidly increasing in South Africa and other settings undergoing epidemiological transitions [12, 13]. Life course interventions rooted in the Developmental Origins of Health and Disease (DOHaD) framework, which emphasizes the importance of exposures starting from the preconception period on future health and development, are potentially an important avenue for the prevention and reduction of these conditions [14–16].

Due to the multifaceted nature of the HeLTI SA intervention [11], fidelity assessment and reporting is important for the interpretation of the trial results. Our qualitative research has indicated that young women living in Soweto (trial setting) face significant difficulties, such as poverty, interpersonal and family-related issues, and the impact of traumatic events [17]. In developing and evaluating a complex, adaptable, and pragmatic intervention in a resource-strapped setting with such social challenges, unique challenges to adherence and fidelity assessment are introduced [18], making fidelity monitoring critical. Moreover, with fidelity assessment guidelines largely being developed in high-income countries, lessons and opportunities arising from a fidelity plan can inform other complex trials in low- and middle-income settings.

We aim to describe the intervention fidelity protocol for HeLTI SA, based on the NIH Behaviour Change Consortium Fidelity framework [19]. This protocol includes the conceptualization of intervention fidelity, specific fidelity strategies, a monitoring plan, and context-specific challenges to ensuring fidelity. We thereby hope to contribute to the reporting of fidelity assessment protocols in low- and middle-income settings and to minimize the gap between the trial and its practical implications for South Africa.

## Methods

### HeLTI SA overview

HeLTI SA is an individual randomized controlled trial, recruiting 6800 young women between 18 and 28 years old through community-based recruitment methods. Recruitment started in October 2019 and is expected to conclude by the end of March 2022 [11]. Participants have no previous diagnosis of cancer, type I diabetes mellitus, or epilepsy. The intervention is delivered by the trained research staff comparable to community health workers, known as “health helpers”, over four phases (preconception, pregnancy, infancy, and early childhood). The intervention aspect of HeLTI was designed to pragmatically inform the South African public health sector, aligning with “real-world” conditions. Therefore, health helpers are similar to community health workers in South Africa in terms of qualification level, caseload, and salary received. The intervention consists of health literacy resource materials, micronutrient supplements, and sessions to support behaviour change using Healthy Conversation Skills (HCS) [11]. In addition, six areas of participants’ health are actively monitored over the course of the intervention, including BMI, haemoglobin (Hb) levels for anaemia status, blood pressure, haemoglobin A1c (HbA1c) for assessing hyperglycaemia, HIV status, and mental health. Health feedback is given based on these measures, and any necessary actions, including referral to clinical care and adjustments in the dose of micronutrient supplements, are taken (see Table 1 for an overview of the intervention components and dose [11]). At-risk intervention participants also have access to a dietitian at least once during each intervention phase. The control arm receives a telephone-based life skills curriculum once a month, in addition to access to standard health care, HIV tests, and pregnancy testing. In the preconception phase, women are followed up for 18 months, or until they conceive and enter the pregnancy phase and continue to the 60-month early childhood trial phase.

**Table 1:** Overview of the intervention components and dose for HeLTI SA arms

**Trial phase**	**Intervention component**				
**Intervention**	**Health literacy resources (*n*, books)**	**Multi-micronutrient supplement (*n*, monthly doses)**	**In-person session and health feedback (*n*, sessions)**	**Telephonic contact (*n*, contact points)**	**Dietitian (for at-risk participants) (*n*, sessions)**
Preconception (18 months)	3	18	3	9	1
Pregnancy (9 months)	1	5	2	3	1
Early childhood (60 months)	1	6	10	20	1
**Control**			**In-person session (life skills), services offered (*n*, sessions)**	**Telephonic contact (life skills) (*n*, contact points)**	
Preconception (18 months)			3	9	
Pregnancy (9 months)			1	3	
Early childhood (60 months)			10	20	

Ethical approval for the HeLTI SA trial and process evaluation was obtained from the Human Research Ethics Committee (Medical) (HREC) of the University of the Witwatersrand, South Africa, with reference numbers M1811111 and M190449. The trial is registered with the Pan African Clinical Trials Registry (https://pactr.samrc.ac.za; identifier: PACTR201903750173871).

### Conceptual framework for intervention fidelity protocol

The overarching conceptual framework for this protocol is the Treatment Fidelity Framework designed by the NIH Behaviour Change Consortium [19], which is used widely across behaviour change research. The framework outlines five main areas for intervention fidelity: study design, provider training, delivery, intervention receipt, and intervention enactment, summarized below.

1. *Study design* refers to the underlying theory and clinical processes and includes the theoretical framework, intended dose for the intervention and control group, and the intended content.
2. *Provider training* refers to the methods to ensure that providers have been satisfactorily trained to deliver the intervention to participants and encompass the specific methods used to train, standardize training, and maintain provider skills throughout the intervention.
3. *Intervention delivery* refers to the methods to ensure the intervention is delivered as specified, including the dose, intervention plan, content, and assessment of non-specific treatment effects.
4. *Intervention receipt* encompasses the degree to which the intervention aligns with the participant’s understanding and their ability to use the skills during the session.
5. *Enactment* refers to the degree to which participants can use the learned skills outside of the intervention sessions and apply them to their own behaviour [19].

**Fig. 1:**
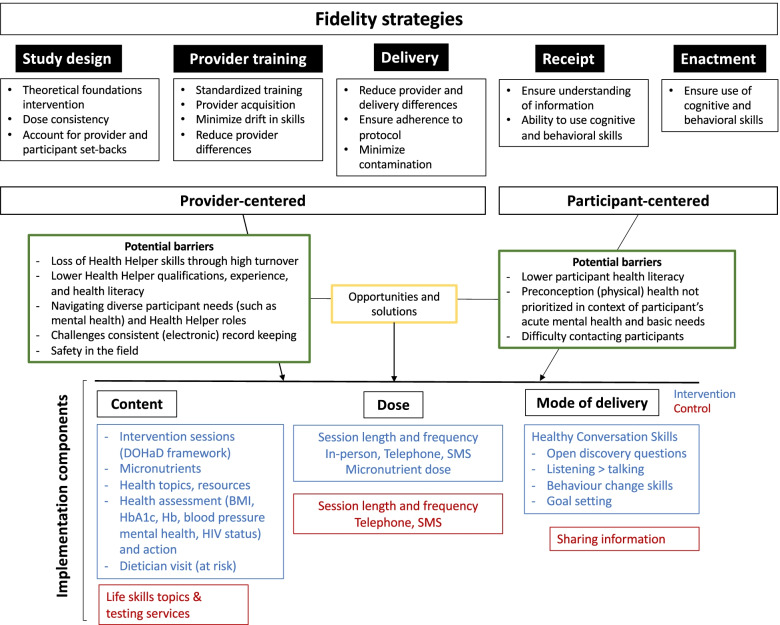
Conceptual framework of fidelity strategies applied to the implementation components of HeLTI SA, outlining the potential barriers

Figure 1 shows the conceptual framework of the Treatment Fidelity Framework adapted to HeLTI SA, with the five components of the NIH fidelity framework and the corresponding strategies as they apply to the implementation components (content, dose, mode of delivery) of the trial. The five fidelity components have been categorized as “provider-centred” (study design, provider training, and delivery) versus “participant-centred” (intervention receipt and enactment), to distinguish between the different potential barriers to implementing and reporting on the fidelity strategies. Both the provider-centred and participant-centred fidelity strategies have the potential to impact the (fidelity of) content, dose, and mode of delivery of the intervention, as described in the individual sections below. The criteria developed to assess the fidelity of the implementation components of the intervention and control group can be found in Additional file 1: Figs. S1 and S2.

## Fidelity strategies and monitoring plan

The below sections outline the strategies for HeLTI SA for each fidelity domain, including the main fidelity strategies employed, the monitoring plan per domain, perceived barriers to fidelity, and possible solutions and opportunities. An overview of the study’s fidelity monitoring plan and data sources can be found in Table 2. Fidelity monitoring, evaluation, and feedback will occur on an ongoing basis throughout the trial using the sources described in Table 2.

**Table 2:** Overview of the study fidelity monitoring plan

*Monitoring tools*	*Areas of fidelity addressed*				
	*Study design*: theoretical framework, intended dose, and intended content	*Provider training*: standardize training and maintain provider skills	*Delivery:* intervention delivered as specified	*Receipt*: participant’s understanding and ability to use the skills during the session	*Enactment:* participants’ ability to use and apply skills in real life
*Observation or recording of intervention sessions and accompanying criteria* (Additional file 1: Figs. S1 and S2) *(1–2 sessions per month per health helper)*	X	X	X	X	X
*(Review of) electronic workflow (activity/contact logs and weekly electronic data dashboards)*	X		X	X	X
*Individual session checklist*		X	X		
*External audit of trial standard operating procedures and trial materials*			X		
*Qualitative evaluation methods (including focus groups and in-depth interviews with intervention participants, health helpers, and control group staff)*		X	X		X
*Health helpers review and monitor progress, understanding, and ability of skills during each session*		X		X	X
*Health helpers reflect on their own use of HCS per session*	X		X		
*Health helpers debrief notes from sessions*			X	X	
*Weekly team debriefs with health helpers*		X	X		
*Quarterly quality assurance reports*	X		X		
*Evaluation following training of each new health helper and maintenance training*		X			
*Participant complaints (as these arise)*		X	X		
*Record of health helper attrition*	X	X			

*HCS* Healthy Conversation Skills

### Fidelity of study design

To evaluate the fidelity of the study design, the following questions can be addressed: *To what extent does the intervention reflect its theoretical foundations? How does the study ensure an equivalent “dose” between and within phases? How does the study ensure the intervention dose is the same across multiple behavioural targets? How does the study plan to address possible intervention setbacks?* [19]

#### Theoretical foundations

DOHaD science informed HeLTI SA’s design. DOHaD considers the impact of environmental factors during critical windows of development (such as intra-uterine exposures) on health, development, and disease risk throughout an individual’s life course [16]. The preconception period has been identified as a promising window for intervention to improve such exposures and subsequently increase long-term population health [20]. HeLTI SA reflects this theoretical foundation by aiming to improve key maternal factors that have been central to DOHaD research, such as body composition, nutrition, and metabolic health, in women from the preconception period through pregnancy and into early childhood, in order to evaluate the impact on childhood obesity, as well as maternal health outcomes.

The behaviour change components of HeLTI SA are grounded in three main theories that, together, reflect the combined importance of context, the individual, and support for behaviour change. Firstly, the theory of planned behaviour suggests that intentions predicate behaviours and that these are determined by factors including personal attitudes, subjective norms, and perceived control; secondly, control theory proposes that behaviour results from minimizing deviation from goals, standards, and ideals; lastly, social cognitive theory suggests an interaction between goal-directed behaviour, personal factors, and environment or social context [11]. HeLTI SA employs Healthy Conversation Skills (HCS), which is based on social cognitive theory and is designed to increase the ability of staff to support behaviour change using more productive conversations through participant empowerment, self-efficacy, and problem solving [21, 22]. Specific techniques inherent to HCS include using open-ended (“discovery”) questions, listening more than talking, and using the HCS SMARTER planning tool (22 ,23). The Specific, Measurable, Action-oriented, Realistic, Timed, Evaluated, Reviewed (SMARTER) planning tool is a component of HCS used to encourage participant’s goal setting for behaviour change targets [22].

The HCS approach is applied during each of the HeLTI SA intervention sessions and is incorporated into the intervention manual for each phase. To evaluate whether participant sessions reflect the theory that HCS is based on, its use is monitored through the completion of a form by health helpers, which includes self-reflection on the use of HCS (e.g. “who did most of the talking?”). This information is monitored by project coordinators through REDCap, the data management system used by HeLTI SA [23]. This tool also allows for the unique information to be captured per participant in a “Notes” section, since the participant-driven nature of HCS can result in non-health-related factors being central to the conversations. Secondly, as a part of the intervention process evaluation, at least 1–2 sessions per month per health helper are observed or recorded by the process evaluation team, which consists of the research staff not involved in intervention delivery. These sessions are evaluated for HCS use through the standardized criteria in Additional file 1: Fig. S1, under “delivery – Healthy Conversation Skills”, and feedback is provided every 2 months.

#### Study design and dose

The intervention dose for HeLTI SA consists of three main components: the health resources, the in-person and telephonic intervention sessions with health helpers, and the micronutrient supplements. The optimal dose is described in detail in the intervention manual/protocol for each intervention phase, and although the number and frequency of each dose component are pre-determined, the duration of individual participant sessions is based on participant needs and is expected to be diverse. The duration of each phase is outlined in the intervention protocol and is fixed, except for the preconception phase which varies depending on the timing of the participant’s potential pregnancy. Moreover, the monthly contact sessions and level of content are consistent across the intervention phases. The study design incorporates methods for electronic monitoring of the length, frequency, and number of contact sessions on REDCap. An overview of the strategies and monitoring plan is provided in Table 3.

**Table 3:** Overview of the study design fidelity strategies and monitoring plan for HeLTI SA

Goal	NIH Description	Strategies used in HeLTI SA	Fidelity monitoring tools for HeLTI SA
Ensure intervention is congruent with relevant theory and practice	Operationalize treatment to optimally reflect theoretical roots and define variables relevant to the “active ingredients” of the intervention	- Health helpers encouraged to use HCS and SMARTER planning in all intervention sessions. - Behaviour change techniques incorporated into the intervention health literacy resources. - Health monitoring, feedback, and action (e.g. referral) are in line with the current practice in South Africa.	- Health helper self-reflection of HCS use is reported on REDCap and monitored by project coordinators. - Several intervention sessions are recorded and evaluated according to a pre-determined checklist.
Ensure a similar dose within the intervention group and across phases	Ensure that intervention “dose” (measured by number, frequency, and length of contact) is adequately described and is similar for each participant and across different phases.	- Length, number, and frequency of contact sessions are outlined in the intervention protocol (Fig. 1). - Intervention duration is based on the four fixed phases outlined in the intervention protocol; the preconception phase length is dependent on participant pregnancy. - Individual session duration is dependent on participant needs and expected to be diverse. - An intervention manual per phase has been developed. - Monthly contact and level of contact are consistent across all intervention phases.	- Project coordinators monitor the frequency and number of intervention sessions via electronic activity log on REDCap. - Notes section is available at the end of the activity log to document and review the unique information about the participant or session. - The health helpers monitor the progress with SMARTER goal planning in each session. -Quarterly quality assurance reports.
Plan for setbacks	Address possible setbacks in implementation (e.g. intervention providers dropping out).	- New health helpers are hired when necessary and are dependent on funding. - Details of the potential suitable future health helper candidates are kept on file.	- Attrition of health helpers is recorded and flagged by human resources and project coordinators.

*NIH* National Institute of Health [19], *HCS* Healthy Conversation Skills

#### Challenges and opportunities in our setting

There are some potential challenges to ensuring fidelity to study the design in our setting. Reaching participants through in-person sessions may be more difficult than telephonic sessions, an issue amplified by reduced in-person contact due to the ongoing COVID-19 pandemic. As a result, the dose of in-person sessions may be disproportionally lower than the telephonic sessions, potentially affecting the effectiveness of HCS and SMARTER planning strategies as discussed in more detail in the “Intervention delivery; Challenges and opportunities in our setting” section. Nevertheless, the use of telephonic sessions allows for a continuation of the intervention despite the onset of the COVID-19 pandemic. Another challenge in terms of planning for intervention setbacks is provider attrition, which could lead to a gap in providers delivering the interventions (see the “Provider training; Challenges and opportunities in our setting” section).

### Provider training

To improve uniformity and quality of intervention delivery, it is essential to train the intervention providers (“health helpers”) in terms of the study protocol, content to delivery to the participants, and new skillsets and to maintain these skills for the duration of the study. The following questions help evaluate the study’s fidelity to provider training: *To what extent and how was training standardized across health helpers? How will skill acquisition be measured? How will skills be trained across health helpers with different backgrounds, training, or skill levels? How will loss or change in skills be minimized within and across health helpers?* [19] (Table 4).

**Table 4:** Overview of the provider training fidelity strategies and monitoring plan for HeLTI SA

Goal	NIH description	Strategies used in HeLTI SA	Fidelity monitoring tools for HeLTI SA
Standardize training	Ensure that training is conducted similarly for different providers	- To ensure providers meet similar a priori criteria and detailed job descriptions are used to recruit health helpers. - Health helpers are trained following their recruitment, in small groups [4, 5], by a core group of trainers. - A detailed training manual with materials is used for training. - HCS training includes role-playing, and the training team was trained by a developer.	- Training session includes a before/after evaluation of skills. - Regular debriefs are held with HHs.
Ensure provider skill acquisition	Train providers to well-defined performance criteria	- Follow-up training on HCS was provided for the HeLTI SA trainers. - Regular problem-solving and debriefing sessions in the form of team meetings. - Certification is not necessary in the South African setting	- Criteria for intervention adherence (Additional file 1) are used to evaluate the intervention and control sessions, generating a score. - Training session includes a before/after evaluation of skills.
Minimize “drift” in provider skills	Ensure that provider skills do not decay over time	- Refresher training for HCS is provided at a standardized time since recruitment. - The project coordinator and researcher are available to provide support and assistance with questions, emergencies, or triggered safety protocols. - Weekly debrief sessions are held, during which questions and concerns can be addressed.	- Observations and review of the recorded sessions are included as a part of the intervention process evaluation. - Qualitative methods are used as part of the process evaluation to assess the participants’ and health helpers’ perceptions of intervention delivery.
Accommodate provider differences	Ensure an adequate level of training in providers of differing skill levels, experiences, or professional backgrounds.	- Consistency in the delivery of intervention components is encouraged and trained. - Strengths of different providers are shared within teams to enhance delivery.	- Observations and review of the recorded sessions are included as a part of the intervention process evaluation.

*NIH* National Institute of Health [19], *HCS* Healthy Conversation Skills

#### Training and skill maintenance

The health helpers are recruited using a detailed job description, ensuring similar skill and experience levels across health helpers. Following employment, health helpers receive standardized training from the HeLTI SA trainers, who consist of researchers and project coordinators trained as HCS trainers with expertise in each intervention phase. Training resources are provided for each phase, such as trial-specific booklets, videos, and relevant resources. For the HCS training component, this includes practice and observation through role-playing during training sessions. Training generally occurs in small groups of 4–6 people, with components covering a period of roughly 28 days. However, due to health helper turnover, training can be completed in phases (rather than 28 consecutive days) and with as few as one health helper per training group. Where possible, the same instructor is used for each group of health helpers. A pilot study describing the implementation of the training method and intervention delivery has been evaluated and reported elsewhere [10]. An official certification of health helpers is not required or provided in the South African setting, but to ensure skill acquisition following training, an assessment of HCS skills is done immediately before and after the training session. This consists of questions requiring the health helper to provide an appropriate HCS-based response to fictitious participant quotations, and responses are subsequently coded using a standardized rubric to determine the degree of improvement since the start of the training and to identify any areas requiring additional attention. This assessment is in addition to the evaluation of the recorded sessions for each health helper, as described above (the “Theoretical foundations” section).

Due to the extensive duration of the complete intervention (up to 87 months), maintaining the health helpers’ skills throughout the intervention is particularly important. Refresher training is provided when required, as determined by continuous process evaluation of intervention sessions. Continued adherence by health helpers to the trained components of HCS is monitored as described under the “Theoretical foundations” section. HeLTI SA’s process evaluation also includes extensive qualitative methods to assess participants’ perceptions of intervention delivery and the presence of non-specific intervention effects originating from specific health helpers. These qualitative methods include focus groups with health helpers, individual in-depth interviews with participants, and session observations and recordings. Any participant complaints, and their relevance to the fidelity of intervention delivery, are reviewed and evaluated by the study researcher and project coordinator. Moreover, weekly debriefing sessions are held during team meetings, where successes or strengths can also be shared with the team, to enhance and increase the uniformity of delivery. Health helpers are also supported by a project coordinator and researchers in case of questions, whenever problem solving is needed, or in the case of emergencies.

#### Challenges and opportunities in our setting

A potential challenge to fidelity for behavioural interventions in general is that there are unavoidable differences between providers, including personal communication style, experience, and interpersonal conduct. In pilot work, health helpers were mostly young women (aged 20–30) having completed secondary education, recruited for their ability to engage and work with the target participants (young women from varying socioeconomic circumstances). For HeLTI SA, as in the South African real-life setting for community health workers, health helpers will not be required to have an official tertiary or health qualification. While past experience within (community) healthcare is an asset, it is not a prerequisite for the health helper position. While these factors reflect a real-life situation relevant to implementation and potential scale-up of the intervention, they may result in greater variation between health helpers in terms of educational background, health literacy, training, or professional experience. The standardized training, training material, and adherence to the study protocol can help to mitigate the differences between individual health helpers.

A second potential challenge to provide training in our setting, as mentioned under the “Fidelity of study design; Challenges and opportunities in our setting” section, is health helper turnover as a result of better-paying job opportunities. This can result in a loss of, rather than increasingly ingrained, skills. Health helper attrition can also result in an increased demand for training of newly hired health helpers. Limited resources within the study team and the need for frequent training sessions with smaller trainee groups (rather than one large group at the beginning of the intervention) may increase the variability in content and rigour of training sessions. There is an opportunity here to develop efficient training processes suitable for our context and the health helper’s knowledge and skills. For example, combining traditional training sessions with pre-recorded training videos for at-home watching, ongoing peer-to-peer training and support, observation of experienced health helpers, and feedback sessions may be more pragmatic and useful in our setting. In addition, recruitment of younger health helpers with less work experience who are more satisfied with the offered salary may help to reduce health helper turnover

### Intervention delivery

Fidelity to intervention delivery refers to the extent to which the intervention is delivered as intended, in a standardized way and according to protocol. The following questions relating to intervention delivery can be addressed: *How will the study measure and control for external or non-specific intervention effects? How can delivery of the intended intervention by providers be ensured? How can differences within intervention groups be reduced? How can adherence to the intervention protocol be ensured?* (Table 5) [19]

**Table 5:** Overview of the intervention delivery fidelity strategies and monitoring plan for HeLTI SA

Goal	NIH description	Strategies used in HeLTI SA	Fidelity monitoring tools for HeLTI SA
Control for provider differences	Monitor and control for participant perceptions of non-specific intervention effects across the intervention group	- Providers are selected for specific characteristics.	- Qualitative methods are used to assess participants’ perceptions of intervention delivery. - Any participant complaints are reviewed by the researcher and project coordinator. - Debrief sessions with health helpers.
Reduce differences within the intervention	Ensure that providers are delivering the same intervention	- The intervention manual developed for each phase is used. - Rather than a script, session checklists on REDCap have been developed to encourage session standardization.	- Electronic activity logs and checklists will be monitored in REDCap by the project coordinator.
Ensure adherence to the intervention protocol	Ensure that the intervention is being delivered in the way in which it was conceived with regard to content and dose	- The intervention manual per phase outlines relevant content. - Session checklists on REDCap guide the intervention content. - Health helpers keep a contact and activity log per participant on REDCap.	- Electronic activity logs and checklists monitored for completion and adherence by the project coordinator. - Monitoring of health assessment outcomes and actions taken on REDCap. - Health helper’s debrief notes and recorded sessions are reviewed by the process evaluation team for non-specific intervention effects, omissions, and intervention delivery. - External audit of standard operating procedures and trial documents. - Quarterly quality assurance reports.
Minimize contamination between groups	Minimize contamination across intervention and control groups, especially when implemented	- HeLTI SA intervention materials and manuals are intervention-specific and not publicly available. - Health helpers are specific to the intervention or control group.	- Observation and recording of sessions are part of the process evaluation.

*NIH* National Institute of Health [19], *HCS* Healthy Conversation Skills

#### Controlling for provider differences

HeLTI SA fidelity strategies aim to reduce and monitor the differences between health helpers that may be present, as described under the "Provider training" section.

#### Reducing differences within intervention and ensuring adherence to protocol

To reduce differences within the intervention and ensure adherence to the protocol, session checklists are used to guide intervention activities and conversation, and intervention materials have been developed for distribution to participants. An exact script is not used for intervention sessions, as this is not compatible with the HCS approach, nor is it realistic in this context. Intervention dose delivery is monitored by the project coordinator through electronic data capturing and workflow using REDCap. This includes a weekly data dashboard and activity log, which allows health helpers to capture the number of sessions conducted vs missed (intervention adherence), whether supplements were delivered, and whether resource materials are received by the participant. This also captures data on the assessment of six areas of health (HIV, HbA1c, Hb, BMI, mental health, and blood pressure) and the relevant actions that are undertaken (such as referral) throughout the intervention. The implementation fidelity criteria in Additional file 1: Figs. S1 and S2 are used to evaluate these records. Health helper debrief notes are evaluated for any notable omissions in the delivery of the intervention. The research team also produces quarterly quality assurance reports for the data that is collected. Moreover, the standard operating procedures (SOPs) and trial documents are externally audited to assure standardization and quality of intervention delivery and data collection.

#### Minimize contamination between the groups

Women in HeLTI SA are individually randomized, since cluster randomization proved to be unfeasible in the pilot trial [10]. The intervention materials are intervention-specific and are not available publicly. Therefore, the risk of contamination is low. While some materials draw on publicly available resources (such as the *Road to Health* booklet, a parent-held birth and development medical record given to each child born in South Africa), the delivery of these by trained health helpers is still unique to the intervention arm. The providers of the control components, trained call centre agents, are also trained on the distinction between the intervention vs control arms of the trial during their training sessions.

#### Challenges and opportunities in our setting

As described in the “Provider training; Challenges and opportunities in our setting” section, a potential challenge for HeLTI SA is attrition of health helpers. There are also a number of challenges that health helpers may experience while delivering the intervention. Firstly, participants can be difficult to trace for appointments, complicating the adherence to the protocol, a difficulty that is amplified by the COVID-19 pandemic. Other methods of contact and frequent reminders for visits, for example, through text messaging and in-person tracing, may help with these challenges. Telephonic sessions, while potentially more practical than in-person visits, have their own challenges in our setting, including participants’ frequent change of phone numbers, unreliable electricity supply, and high data costs for Internet-based communications. Furthermore, participants may not have access to a private place for telephonic sessions in which potentially sensitive and private topics are discussed, emphasizing the need for face-to-face sessions for the effective delivery of HCS.

Since a tertiary degree or specific past experience is not required, the delivery may be impacted by limited health literacy amongst health helpers. However, as described in the “Provider training” section, provider training is used to minimize such differences amongst health helpers. Lastly, formative research from the pilot trial has indicated that health helpers will likely encounter varying participant needs, which in our setting are not always health-related or relevant to the intervention manual [10, 17], as discussed in more detail under the “Intervention receipt and enactment; Challenges in our setting” section.

### Intervention receipt and enactment

The final two intervention fidelity components, intervention receipt and enactment, are centred around the participant rather than the provider and are therefore challenging to uphold and monitor in many settings. Intervention receipt refers to the extent to which the participant can understand and perform intervention-related skills and cognitive strategies during delivery, whereas intervention enactment refers to the extent to which such skills and strategies can be performed in the intended real-life situations. In a longitudinal trial with behaviour change components such as HeLTI SA, the outcome is dependent on the extent of participant receipt and enactment. Questions for evaluating intervention receipt include the following: *How can the participant’s understanding of the provided information be verified? How can the participant’s ability to use cognitive and behavioural skills taught during the intervention be verified? How can issues that interfere with receipt be addressed?* (Table 6) [19].

**Table 6:** Overview of the intervention receipt fidelity strategies and monitoring plan for HeLTI SA

Goal	NIH description	Strategies used in HeLTI SA	Fidelity monitoring tools for HeLTI SA
Ensure participant comprehension	Ensure that participants understand the information provided in the intervention	- HCS, based on questions and discussion of content, underpins the intervention sessions. - The intervention is based on achievement-based goals using the SMARTER planning tool. - Activity logs are completed to guide the subsequent sessions.	- Health helpers review and monitor the understanding of the intervention by reviewing SMARTER goals and discussing barriers to progress. - Review of electronic activity logs.
Ensure participant ability to use cognitive and behavioural skills	Ensure that participants are able to use the cognitive and behavioural skills taught in the intervention	- Health helpers guide participants on SMARTER goals, review actual or potential barriers, and assess confidence in achieving SMARTER goals. - Exploratory questions during sessions guide participants to problem-solve barriers and identify solutions.	- Health helpers monitor participants’ ability during intervention sessions. - Health helpers’ debrief notes and recorded sessions are reviewed by the process evaluation team.

*NIH* National Institute of Health [19], *HCS* Healthy Conversation Skills

Questions to address intervention enactment include the following: *How can participant use of cognitive and behavioural skills taught in the intervention in the appropriate real-life situations be verified? How can issues that interfere with enactment be addressed?* (Table 7) [19].

**Table 7:** Overview of the intervention enactment fidelity strategies and monitoring plan for HeLTI SA

Goal	NIH description	Strategies used in HeLTI SA	Fidelity monitoring tools for HeLTI SA
Ensure participant use of cognitive skills	Ensure that participants actually use the cognitive skills provided in the intervention in appropriate life settings	- Health helpers guide participants on SMARTER goals, monitor the progress, and review the barriers to the use of cognitive skills. - During telephone sessions, health helpers discuss the ongoing use of new cognitive skills underpinning SMARTER goals.	- Observation and recording of sessions to review the use of cognitive skills. - Qualitative methods to assess participants’ perceptions of intervention and changes they have implemented as a result.
Ensure participant use of behavioural skills	Ensure that participants actually use the behavioural skills provided in the intervention in appropriate life settings	- Monthly contact encourages adherence to and allows for monitoring of participant behavioural changes. - Contact and activity log on REDCap are filled in by health helper. - Health helpers guide participants on SMARTER goals, monitor the progress, and review the barriers to the use of behavioural skills.	- Observation and recording of sessions to review the behavioural changes reported by participants. - Monitoring electronic log of monthly contact on REDCap by the project coordinator. - Qualitative methods to assess participants’ perceptions of intervention and changes they have implemented as a result.

*NIH* National Institute of Health [19]

#### Ensuring participant comprehension and ability to use skills (receipt)

Through the use of HCS and the SMARTER planning tool, health helpers use exploratory questions and discuss content with participants while setting achievement-based goals. Participant input is therefore central to the intervention sessions, allowing health helpers to monitor and adjust the intervention to the participant’s understanding and specific goals. Electronic logs of the session are completed to guide content for the subsequent sessions and to ensure it is appropriate for individual participants’ needs and understanding. By discussing the potential barriers, guiding participants to identify solutions, and assessing their confidence in achieving SMARTER goals, health helpers are also able to ascertain participant ability to use the skills. Lastly, participant receipt of the intervention is evaluated by the process evaluation team, consisting of researchers and of research staff not actively involved in other parts of the trial, through a review of a selection of recorded sessions, qualitative interviews with participants, and health helper debrief notes.

#### Ensuring participant use of skills (enactment)

Observation of participants outside of intervention sessions is not in the scope of the HeLTI study design. Using the SMARTER planning tool during in-person and telephonic sessions, health helpers monitor the progress of goals and review barriers to use throughout the duration of the intervention. The regular monthly nature of contact sessions encourages adherence to behavioural changes and allows health helpers to keep track using the contact and activity log on REDCap. Lastly, enactment is monitored through qualitative methods, such as in-depth participant interviews and session observations, to assess participant perception of the intervention and perceived (cognitive and behavioural) changes resulting from the intervention.

#### Challenges in our setting

Fidelity to both receipt and enactment of the intervention is dependent on participant circumstances, needs, and background. Low health literacy is a concern in low- or middle-income settings [24, 25], and amongst young women in our setting, formative work has shown low health literacy around preconception health [17]. This may hinder participant understanding and ability to use aspects of the intervention. Additionally, it may increase the extent to which health helpers provide information rather than using HCS strategies such as listening more than talking, making it more difficult to monitor both participants’ understanding and their use of the intervention skills.

Participants in our setting have been found to live with pressing social and economic challenges, which may overshadow the importance of a health-related intervention (receipt) and reduce participants’ agency and autonomy to enact change (enactment) [17, 26]. In addition, access to healthcare following clinical referrals arising from participant health assessment may be limited by systemic resource constraints on the healthcare system in South Africa. Both health literacy levels and the systemic issues faced by participants may make it more difficult to set health-related goals, address intervention materials around health, monitor the progress of goals, and overcome barriers or find solutions. However, taking the time to address participants’ needs and establishing trust could, in the long run, make intervention delivery more effective, although it may take longer than expected to reach health-related targets. Lastly, varying levels of record and note keeping by the health helpers is a limitation noted in the pilot work of the trial, which could hinder the ability to monitor receipt and enactment. Additional training and encouragement of record keeping in weekly debriefs will be employed to improve record keeping.

## Discussion

Based on the NIH BCC conceptual guidelines for fidelity intervention improvement and monitoring, this paper describes the intervention fidelity protocol and monitoring plan for HeLTI SA, the ongoing randomized controlled trial investigating the impact of a complex, multi-phase intervention on maternal and child health in Soweto, South Africa. The aim of establishing and standardizing a fidelity protocol is to increase the ability to reliably draw conclusions about the effectiveness of the intervention in question and its underlying theory. However, the development of the protocol for our complex behaviour change intervention has also highlighted the challenges in our setting.

In low- and middle-income settings, where reporting of fidelity has been found to be inconsistent [4], transparency around the challenges involved with adhering to a standardized fidelity approach is needed. The implications could include a decreased ability to apply trial findings to policy and practice. While such challenges will vary between settings, increased reporting of both fidelity strategies and the challenges involved can help to highlight common issues and foster the development of solutions. Potential fidelity challenges highlighted by the formative work for HeLTI SA include high health helper turnover, lack of higher education qualifications amongst health helpers, difficulty tracing participants for sessions, and barriers to effectively implement HCS and other intervention components. These barriers include the need to prioritize participants’ non-health-related challenges, low health literacy amongst participants, and barriers to the use of healthcare in case of a clinical referral. The COVID-19 pandemic may additionally exacerbate some of these challenges, such as the tracing of participants and the social and economic challenges faced by participants.

Since HeLTI SA has been designed to pragmatically inform the local South African healthcare landscape, these challenges to fidelity are also important to consider in terms of the feasibility and scalability of the intervention, and other similar interventions, in real-life settings. Some proposed or implemented solutions include encouraging health behaviour change in the context of the participant’s pressing life circumstances and adapting health helper training strategies to be feasible yet thorough in the context of high turnover and limited resources. Fidelity to the intervention protocol versus the adaptability needed to implement such solutions may seem mutually exclusive. However, adaptations based on pilot findings to improve delivery, adherence, and participant engagement can be made while upholding the key functional components of the intervention. This concept of “functional fidelity” has been described for the implementation of complex behaviour change interventions [27, 28], and fidelity monitoring strategies increase the ability to record and evaluate both intended and unintended variations in the intervention [29]. A recent update of the MRC Framework for the Development and Evaluation of RCTs for Complex Interventions, first published in 2000 [30], also emphasizes the need for a flexible model for intervention development and implementation [29, 31]. However, guidelines for researchers attempting to navigate intervention fidelity challenges, particularly for interventions in low- and middle-income settings, are lacking.

In conclusion, the five NIH BCC fidelity components were used to develop a fidelity protocol and monitoring plan for HeLTI SA and to improve the ability of investigators to establish whether the intervention is being implemented as intended. In addition, recognizing challenges to both ensuring and monitoring intervention fidelity allows researchers to transparently find solutions that prevent compromising the study’s ability to draw reliable conclusions. The development and sharing of fidelity protocols and associated challenges, particularly in low- and middle-income settings, can help future researchers to develop fidelity strategies and monitoring plans despite the presence of context-specific challenges.

## Trial status

Fidelity protocol version: 1.0; Date: December 2021. Recruitment for HeLTI SA (*Bukhali*) started in October 2019 and is expected to conclude by the end of August 2022.

## Supplementary Information

**Additional file 1: Fig. S1.** Criteria checklist to assess fidelity of the implementation components for the intervention arm of the trial. **Fig. S2.** Criteria checklist to assess fidelity of the implementation components for the control arm of the trial.

## Data Availability

Not applicable (study protocol).

## Abbreviations

DOHaD: Developmental Origins of Health and Disease
Hb: Haemoglobin
HbA1c: Haemoglobin A1c
HCS: Healthy Conversation Skills
HeLTI SA: Healthy Life Trajectories Initiative South Africa
HREC: Human Research Ethics Committee
LMIC: Low- and middle-income country
NIH BCC: National Institutes of Health Behaviour Change Consortium
RCT: Randomized controlled trial
SOP: Standard operating procedure
