# Supplementary material for: A protocol for monitoring fidelity of a preconception-life course intervention in a middle-income setting: the Healthy Life Trajectories Initiative (HeLTI), South Africa
Source: Trials. 2022 Sep 6;23:758. doi: 10.1186/s13063-022-06696-w (PMC9449293; doi:10.1186/s13063-022-06696-w)
Supplement: Supplementary file 1 — Additional file 1: Fig. S1. Criteria checklist to assess fidelity of the implementation components for the intervention arm of the trial. Fig. S2. Criteria checklist to assess fidelity of the implementation components for the control arm of the trial. [file 13063_2022_6696_MOESM1_ESM.pdf]

Figure S1: criteria checklist to assess fidelity of the implementation components for the intervention arm of the trial.

Implementation components	Level of fidelity		
**INTERVENTION**	**Low** (score 1)	**Medium** (score 2)	**High** (score 3)
**Dose**			
Frequency of sessions / contacts (face-to-face / telephonic / SMS)*	Every 8 weeks or more	Every 6-7 weeks	Every 4-5 weeks
Length of sessions (face-to-face / telephonic), including catch up	Too short to deliver session/s	Adequate to deliver session/s	More than adequate to deliver session/s
On track with sessions**	Catching up 4 or more sessions	Catching up 2-3 sessions	On track with sessions / catching up 1 session
**Delivery – Healthy Conversation Skills**			
Open Discovery Questions	Hardly / did not use ODQs	Used ODQs occasionally	Used ODQs extensively
Listening vs giving information	Mostly gave information rather than listened	Roughly even balance between listening and giving information	Mostly listened rather than give information
Behaviour change (doesn't have to be a health behaviour change)	Did not address behaviour change	Addressed behaviour change to some extent	Addressed behaviour change extensively
Goal setting (doesn't have to be a health goal)	Did not address goal setting	Addressed goal setting to some extent	Addressed goal setting extensively
**Content**			
Relevant information for the sessions (depending on content provided for the session)	Did not cover relevant information at all	Covered some relevant information	Covered relevant information well
Relevant services (depending on participants' needs)	Did not offer services when they should have	Offered services, but did not provide adequate details / Offered some but not other services	Offered services and provided adequate details

*From REDCap records; all other data from observation / listening **Note session number

Total score (max 27): ☐

Figure S2: criteria checklist to assess fidelity of the implementation components for the control arm of the trial.

Implementation components	Level of fidelity		
**CONTROL**	**Low** (score 1)	**Medium** (score 2)	**High** (score 3)
**Dose**			
Frequency of sessions / contacts (face-to-face / telephonic / SMS)*	Every 8 weeks or more	Every 6-7 weeks	Every 4-5 weeks
Length of sessions (face-to-face / telephonic), including catch up	Too short to deliver session/s	Adequate to deliver session/s	More than adequate to deliver session/s
On track with sessions**	Catching up 4 or more sessions	Catching up 2-3 sessions	On track with sessions / catching up 1 session
**Delivery**			
Professional (e.g. respectful, protects confidentiality)	Often unprofessional during the conversation	Mostly professional during the conversation	Professional throughout the conversation
Answers participant's questions (or commits to finding out more information)	Does not answer questions / discourages questions	Answers most of participant's questions	Answers all participant's questions
Helps participant feel comfortable	Makes participant feel uncomfortable	Makes participant feel mostly comfortable	Makes participant feel comfortable at all times
Non-judgemental	Judgemental of participant	Mostly non-judgemental	Consistently non-judgemental
**Content**			
Relevant information for the sessions (depending on content provided for the session)	Did not cover relevant information at all	Covered some relevant information	Covered relevant information well
Relevant services (depending on participants' needs)	Did not offer services when they should have	Offered services, but did not provide adequate details / Offered some but not other services	Offered services and provided adequate details

*From REDCap records; all other data from observation / listening **Note session number

Total score (max 27): ☐
